# *Trichomonas vaginalis* Antimicrobial Drug Resistance in 6 US Cities, STD Surveillance Network, 2009–2010

**DOI:** 10.3201/eid1806.111590

**Published:** 2012-06

**Authors:** Robert D. Kirkcaldy, Peter Augostini, Lenore E. Asbel, Kyle T. Bernstein, Roxanne P. Kerani, Christie J. Mettenbrink, Preeti Pathela, Jane R. Schwebke, W. Evan Secor, Kimberly A. Workowski, Darlene Davis, Jim Braxton, Hillard S. Weinstock

**Affiliations:** Centers for Disease Control and Prevention, Atlanta, Georgia, USA (R.D. Kirkcaldy, P. Augostini, W.E. Secor, K.A. Workowski, D. Davis, J. Braxton, H.S. Weinstock);; Philadelphia Department of Public Health, Philadelphia, Pennsylvania, USA (L.E. Asbel),; San Francisco Department of Public Health, San Francisco, California, USA (K.T. Bernstein);; Public Health–Seattle and King County, Seattle, Washington, USA (R.P. Kerani);; Denver Public Health Department, Denver, Colorado, USA (C. Mettenbrink);; New York City Department of Health and Mental Health, New York, New York, USA (P. Pathela);; Jefferson County Department of Health, Birmingham, Alabama, USA (J.R. Schwebke);; University of Alabama, Birmingham (J.R. Schwebke);; Emory University, Atlanta (K.A. Workowski)

**Keywords:** Trichomonas vaginalis, drug resistance, metronidazole resistance, tinidazole resistance, antimicrobial drug resistance, sexually transmitted diseases, protozoa, US cities

## Abstract

Nitroimidazoles (metronidazole and tinidazole) are the only recommended drugs for treating *Trichomonas vaginalis* infection, and previous samples that assessed resistance of such isolates have been limited in geographic scope. We assessed the prevalence of in vitro aerobic metronidazole and tinidazole resistance among *T. vaginalis* isolates from multiple geographic sites in the United States. Swab specimens were obtained from women who underwent routine pelvic examinations at sexually transmitted disease clinics in 6 US cities. Cultured *T. vaginalis* isolates were tested for nitroimidazole resistance (aerobic minimum lethal concentration [MLC] >50 µg/mL). Of 538 *T. vaginalis* isolates, 23 (4.3%) exhibited low-level in vitro metronidazole resistance (minimum lethal concentrations 50–100 µg/mL). No isolates exhibited moderate- to high-level metronidazole resistance or tinidazole resistance. Results highlight the possibility that reliance on a single class of antimicrobial drugs for treating *T. vaginalis* infections may heighten vulnerability to emergence of resistance. Thus, novel treatment options are needed.

## Abstract 2

Such isolates should undergo drug susceptibility testing periodically to detect emerging resistance.

Trichomoniasis, caused by *Trichomonas vaginalis,* is one of the most common nonviral sexually transmitted diseases (STDs): annually, ≈248 million incident cases occur worldwide, and ≈7.4 million cases occur in the United States (*1**,**2*). The estimated US prevalence of *T. vaginalis* infection is 3.1%, with a higher prevalence among black women and women of low socioeconomic status than among other women (*3*). Trichomoniasis is a frequent cause of vaginitis and can contribute to premature rupture of membranes during pregnancy, preterm birth, low birth weight, and may facilitate HIV acquisition (*4**–**7*).

The Centers for Disease Control and Prevention (CDC) (Atlanta, Georgia, USA) STD Treatment Guidelines recommends the use of a 5-nitroimidazole antimicrobial agent, either metronidazole or tinidazole, for the treatment of *T. vaginalis* infection (*8*). Metronidazole has been the mainstay of treatment for several decades; however, tinidazole has better in vitro activity and is well tolerated (*8*).

The reliance on a single drug class for treating *T. vaginalis* infections may be problematic if resistance to nitroimidazole becomes widespread in *T. vaginalis* strains. Three small studies that examined the prevalence of in vitro resistance in the United States have been conducted during the past 15 years, but they were limited in geographic scope (*9**–**11*). Our objective was to assess the prevalence of in vitro aerobic metronidazole and tinidazole resistance among a broad sample of *T. vaginalis* isolates from multiple geographic sites in the United States.

## Methods

Demographic data and *T. vaginalis* isolates were collected from women attending 6 STD clinics participating in the STD Surveillance Network (SSuN). SSuN is a sentinel site surveillance network which, through the implementation of common protocols for collecting, reporting, and analyzing enhanced surveillance data, aims to improve the capacity of national, state, and local STD programs to detect, monitor, and respond rapidly to trends in STDs. *T. vaginalis* specimens from women undergoing physical examinations were systematically collected, either consecutively or on selected days of the week, during 2009 and 2010, in the following cities: Birmingham, Alabama (n = 80 viable isolates submitted); Denver, Colorado (n = 99); New York, New York (n = 93); Philadelphia, Pennsylvania (n = 103); San Francisco, California (n = 85); and Seattle, Washington (n = 96). Two sites (Seattle and San Francisco) restricted participation to symptomatic women. (Because the data were obtained through a surveillance activity, CDC did not to require human subjects review.)

Data regarding patient’s demographic characteristics, pregnancy status, presence or absence of symptoms suggestive of trichomoniasis (vaginal discharge, odor, or itching), prior trichomoniasis diagnosis in the preceding 12 months, and presence or absence of vaginal discharge on physical examination were abstracted from medical records. Vaginal secretions were collected by using a sterile Dacron swab during the pelvic examination, and the swab was used to inoculate the InPouch TV (BioMed Diagnostics, San Jose, CA, USA) culture media, according to manufacturer’s specifications. Before inoculation, the InPouch TV culture medium was stored at room temperature (18°C–25°C) in a horizontal position away from direct sunlight. Inoculated InPouch TV cultures were incubated at 35°C–37°C for 24–96 hours. A culture was considered positive if at least 1 trichomonad was observed by microscopy. Positive cultures were transported to the Division of Parasitic Diseases and Malaria Laboratory (CDC) by overnight express mail. Upon arrival, parasites were incubated in Diamonds TYM (typticase, yeast extract, and maltose medium) at 37°C until axenic cultures were obtained.

Isolates were assayed for metronidazole and tinidazole susceptibility under aerobic conditions, according to the method developed by Meingassner and Thurne using serial dilutions of drug concentrations from 0.2 to 400 µg/mL (*12*). The minimum lethal concentration (MLC) was the lowest dilution at which no motile trichomonads could be observed from an isolate assay. Isolates were tested in triplicate, and the assay was repeated twice. Control strains were CDC 085 (resistant) and CDC 520 (sensitive). If results differed, the modal result was used. Low-level resistance was defined as aerobic MLC 50–100 µg/mL, moderate-level resistance as 200 µg/mL, and high-level resistance as >400 µg/mL (*13*).

If multiple isolates were submitted from a single patient, we included the first submitted isolate in the analytic dataset. We compared median MLCs of metronidazole and tinidazole by using the Wilcoxon matched pairs signed rank test to account for intra-isolate correlation. We assessed the prevalence of metronidazole and tinidazole resistance among isolates and compared the prevalence of resistance to each agent by geographic site by using the χ^2^ test. We compared median metronidazole MLCs by geographic site by using the Wilcoxon rank sum test. Demographic and clinical data for women infected with a resistant isolate (metronidazole or tinidazole MLC >50 µg/mL) were compared with data for women infected with a susceptible isolate by using χ^2^ or Fisher exact test for dichotomous data and *t* test for continuous data. p values were 2-tailed and considered significant at p<0.05. Analyses were conducted by using SAS version 9.2 (SAS Institute, Cary, NC, USA).

## Results

From April 6, 2009, through November 17, 2010, a total of 560 evaluable vaginal swab specimens were submitted from 538 women (range per woman, 1–3 swab specimens). Of these women, the median age was 28 years (range 13–67 years); 71% were African-American, 11% were non-Hispanic white, 11% were Hispanic or Latina, and 5% were of other race/ethnicity. Race/ethnicity data were missing for 2%. At least 1 previous episode of trichomoniasis was reported by 39% of women who submitted samples. Three percent of the women were pregnant, and none were HIV infected. Symptoms consistent with trichomoniasis (vaginal discharge, odor, or pruritus) were reported by 77% of women. By site, 80 (14.9%) women were from Birmingham, 94 (17.4%) were from Denver, 92 (17.1%) were from New York, 103 (19.1%) were from Philadelphia, 82 (15.2%) were from San Francisco, and 87 (16.2%) were from Seattle.

Of 538 isolates, the median MLC of metronidazole (3.1 µg/mL) was higher than the median MLC of tinidazole (0.8 µg/mL) (p<0.001) (Figure). The prevalence of low-level metronidazole resistance was 4.3% (95% CI, 2.7%–6.4%). No isolates exhibited moderate- to high-level metronidazole resistance; all isolates were susceptible to tinidazole. The prevalence of metronidazole resistance did not vary significantly by geographic site: Birmingham, 1.3% (95% CI, 0.1%–6.8%); Denver, 7.5% (95% CI, 3.1%–14.7%); New York, 2.2% (95% CI, 0.3%–7.6%); Philadelphia, 3.9% (1.1%–9.7%); San Francisco, 4.9% (95% CI, 1.3%–12.0%); and Seattle, 5.8% (95% CI, 1.9%–12.9%). The median metronidazole MLC among isolates from Birmingham (3.1 µg/mL [range 0.4–50 µg/mL]) was lower than among isolates from Seattle (6.3 µg/mL [range 0.4–100 µg/mL]; p = 0.043); otherwise no significant differences in median MLC by site were detected. We did not find significant differences between women infected with a metronidazole-resistant strain and women infected with a metronidazole-susceptible strain in terms of age, race/ethnicity, pregnancy status, symptom status, or having a previous diagnosis of trichomoniasis.

**Figure:**
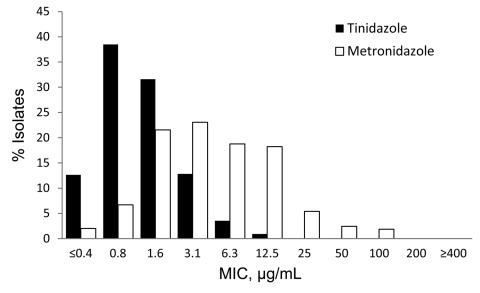
Distribution of minimum lethal concentrations (MLCs) of tinidazole and metronidazole, STD Surveillance Network, 2009–2010 (n = 538). Susceptibility to metronidazole and tinidazole are defined as MLC <25 μg/mL, low-level resistance as MLC 50–100 μg/mL, moderate-level resistance as MLC 200 μg/mL, and high-level resistance as MLC >400 μg/mL.

Sixteen women submitted 2 evaluable isolates, and 1 woman submitted 3 isolates. Among these 17 women, the median duration between sample collection was 128 days (range 2–392 days). Three women submitted the second specimens within 30 days of the initial submission (initial MLCs of metronidazole: 0.8–3.1 µg/mL), 3 within 60 days (initial MLCs 1.6–6.3 µg/mL), and 1 within 90 days (initial MLC 0.8 µg/mL). Data on sexual re-exposure were available for only 1 of these 7 women. For initial isolates from 2 women, MLCs of metronidazole were >12.5 µg/mL (50 µg/mL in both cases). In 1 case, the second isolate was collected 158 days after the first, and the woman reported 12 sex partners in the preceding 3 months. In the other case, the second isolate was collected 308 days later, and the MLC of metronidazole for this strain was 0.8 µg/mL.

## Discussion

To our knowledge, this study is the first multisite evaluation conducted to assess the prevalence of in vitro *T. vaginalis* resistance in the United States. Although metronidazole has been used to treat *T. vaginalis* infections for ≈40 years, we found a low prevalence of in vitro metronidazole resistance. MLCs of tinidazole were lower than MLCs of metronidazole, and we did not detect tinidazole resistance.

The prevalence of in vitro metronidazole and tinidazole resistance is consistent with previously published US estimates. Three studies conducted in the southeastern United States among women attending STD or gynecology clinics from 1997 through 2005 found a metronidazole-resistance prevalence of 2.4%–9.5% (*9**–**11*). Most metronidazole-resistant isolates in these studies exhibited low-level resistance. In 2 of these studies tinidazole resistance also was tested: Krashin et al. did not detect tinidazole resistance (*11*), and Schwebke and Barrientes detected 1 isolate (0.6%) that exhibited low-level tinidazole resistance (MLC 50 µg/mL) among the 178 isolates tested (*10*). Among 91 isolates collected in Spain during 1995 and 1999, 2.2% exhibited low-level resistance to metronidazole (*14*). A small study conducted among women from Papua New Guinea found 21 (91%) of 23 studied isolates had MLCs of metronidazole of >50 µg/mL, including 4 (17%) with MLCs of 200 µg/mL (*15*). However, the sampling method used to enroll women was not described and may not have been systematic, thus substantially limiting the ability to estimate the population-level prevalence of resistance. Investigators in the United Kingdom reported that 1.7% of women treated for trichomoniasis during 1998–2002 appeared to have not responded to treatment and denied re-exposure; in vitro susceptibility data were not available (*16*).

Inconsistency does exist between in vitro susceptibility results and clinical outcomes of treatment, particularly for infections with low-level in vitro resistance. Clinical resistance and treatment failure have occurred with *T. vaginalis* isolates for which MLCs of nitroimidazoles were as low as 12.5 µg/mL, and treatment success has occurred in infections with *T. vaginalis* isolates for which MLCs of nitroimidazoles were 100–200 µg/mL (*13*). In general, however, elevated MLCs are associated with a greater likelihood of treatment failure. A recent evaluation of the utility of susceptibility testing in women for whom clinical treatment has failed found that treatment recommendations based on susceptibility results may have a beneficial role in informing the clinical management of some women with persistent infection (*17*). *T. vaginalis* susceptibility testing is not available routinely; such testing should be conducted by a qualified laboratory and is available at CDC (1–800-CDC-INFO).

As a cross-sectional evaluation of in vitro antimicrobial drug susceptibility, the study was not designed to detect clinical treatment failures. Multiple isolates were collected from 17 women. However, we did not systematically collect data on sexual re-exposure after treatment or adherence, so we were not able to determine whether any of these cases resulted from treatment failure. The MLCs of metronidazole for the initial isolates were low, suggesting that clinical resistance was unlikely. In 2 cases, the initial isolate exhibited low-level resistance (MLC of metronidazole 50 µg/mL) and a second isolate was later collected. Both of these cases were probably re-infections.

For isolates in our study, MLCs of tinidazole were lower than those of metronidazole, which supports the idea that tinidazole should be prescribed for patients whose infections do not respond clinically to metronidazole. This finding is consistent with results of previous studies which showed that tinidazole had better in vitro activity than metronidazole at similar molar concentrations (*18*). Tinidazole has a longer serum half-life than metronidazole and exhibits good tissue penetration (*19*), yet is more expensive than metronidazole. Although tinidazole and metronidazole are the only nitroimidazoles available in the United States, ornidazole, tenonitrazole, and nimorazole are available in Europe and could be alternatives to metronidazole. These agents are of the same drug class as metronidazole, however, and the emergence of clinically notable nitroimidazole resistance would be expected to adversely influence the treatment effectiveness of each of these agents.

This study had several limitations. First, the sample was limited to women attending STD clinics participating in SSuN; thus, our findings are not representative of the general population. In addition, symptomatic women were likely to have been overrepresented because participating women were seeking care in STD clinics, and 2 of the sites sampled only symptomatic women. Also, although we believe this is the largest study of its kind, the sample size may not have been large enough to detect significant differences across sites, nor to detect tinidazole resistance or high-level metronidazole resistance. That we did not detect isolates with such resistance suggests that its prevalence in this population is low. CDC occasionally receives isolates that are highly resistant to metronidazole or tinidazole, however (W.E. Secor, pers. comm.).

Although the prevalence of resistance is currently low in the United States, reliance on a single class of antimicrobial drugs heightens vulnerability if clinical *T. vaginalis* nitroimidazole resistance becomes widespread. Market forces alone are unlikely to spur the development of new anti-trichomonal drugs. Further evaluation of existing compounds and development of novel systemic treatment options are needed, and efforts to promote and support antimicrobial drug development and evaluation are warranted.

In summary, we found a 4% prevalence of low-level metronidazole resistance among *T. vaginalis* isolates from women attending several STD clinics throughout the United States. Periodic sentinel surveillance evaluations of *T. vaginalis* antimicrobial drug susceptibility should be carried out to monitor the possible emergence of resistance.
